# Experiences and contextual practices of family-centered care in Ghanaian nicus: a qualitative study of families and clinicians

**DOI:** 10.1186/s12913-022-08425-0

**Published:** 2022-08-17

**Authors:** Alhassan Sibdow Abukari, Angela Kwartemaa Acheampong, Lydia Aziato

**Affiliations:** 1 School of Nursing, Wisconsin International University College-Ghana, P.O Box LG, Accra, Ghana; 2 University of Health and Allied Health Sciences, Ho, Ghana

**Keywords:** Family-centered care practices, Neonatal intensive care units, Families, Clinicians, Ghana, Exploratory descriptive design

## Abstract

**Background:**

Families, whether at home or at the hospital, should be a vital part of newborn care. However, most families are excluded from hospital care, particularly in neonatal intensive care units (NICUs). This is incompatible with the concepts of Family-Centered Care (FCC) and may compromise care continuity and family satisfaction following discharge from neonatal intensive care facilities. The purpose of this study was to examine FCC practices in Ghanaian neonatal intensive care units and provide the experiences and contextual practices of FCC from the perspectives of families and clinicians.

**Methods:**

The study qualitatively examined the contextual practices of FCC from the perspectives of families and clinicians in neonatal intensive care units using an exploratory descriptive design. With the help of MAXQDA software, 36 transcripts were generated and their contents were analyzed.

**Results:**

Contextual practices of FCC, family experiences of FCC and clinician experiences of FCC emerged as three main categories from the data. Respect and dignity, culture and religion and a multidisciplinary approach were the contextual practices. Emotional stress, lack of information and coping strategies were all common family experiences. Support, counseling, education and financial problems have all been experienced by clinicians.

**Conclusions:**

Shared decision-making, counseling and education, as well as respect/dignity amongst clinicians, managers and families using a multidisciplinary approach are the fundamental concepts of FCC approach in Ghana. Acceptance and integration of FCC approach into neonatal intensive care units may reduce the burden of care as well as improve the quality of care. Further studies are needed to map out strategies and interventions for the integration of FCC into intensive care units.

## Background

Globally, Family-Centered Care (FCC) is a well-known healthcare approach for children and their families [1–4]. It has over the past decades been touted as the new paradigm by paediatricians, especially in the developed world [4–7]. The Institute of Patient and Family-Centered Care (IPFCC) has identified four main concepts of it including respect/dignity, communication, participation and collaboration [1]. FCC is an approach to paediatric and child health care emphasizing respective collaborative partnerships between healthcare professionals and families of sick children in the paediatric health setting to meet their physical, emotional, social, and developmental needs [8–10]. When clinicians accept and implement FCC in neonatal care, it can promote parental satisfaction and shorten the length of stay in the neonatal intensive care unit (NICU) [11, 12]. Yet, its application in the intensive care units (ICU) is faced with several challenges due to contextual preferences across the world.

The practice of FCC in many countries has been reported. For instance, in Asia, the main concepts adopted were support, visual information, collaboration, partnerships and mutual trust [13–15]. The fundamental concepts of FCC on the American continent are information sharing (education and communication), participation (parental involvement), respect and dignity and shared decision-making [16, 17]. Similarly, in Sweden, the training of nurses and physicians improved their support for parents to participate in the care of their children and mentorship to junior clinicians in the ICU was used to appreciate families as experts in the care of children [18]. Norway employed respect and empathy to yield family satisfaction [11], whilst Denmark used family support to address the barriers of FCC [19]. In South Africa, they employed family involvement and the provision of adequate information for infants and families to gain the satisfaction of parents in the ICU [20]. Flexible visiting times were allowed for parents to connect with their hospitalized children in Tanzania [21]. Apart from Europe and the United States which utilize and implement these four concepts, other parts of the world have implemented portions of them based on what works in their context. In the case of Africa, little or no studies exist on FCC in the NICU, especially in Western Africa and Ghana. These findings in the intensive care literature cement the argument for contextualizing the practices of FCC as different settings adopt what works best per their context.

Furthermore, what is also evident in these studies is that the findings are largely emanating from families’ perspectives, albeit a few from clinicians’ perspectives. Meanwhile, the involvement of fathers, mothers, nurses, midwives, doctors and health managers in one study will broaden the contextual practices and appreciation of FCC in any setting. There is, therefore, a need to extensively explore the practices and experiences of both families and clinicians to gain a broader understanding of the contextual practices of FCC. In Ghana, the practices of FCC in the NICUs are poorly documented and hence its contextual practices and the experiences of families and clinicians are vague.

The primary purpose of this study was, therefore, to qualitatively study the practices of FCC in the NICU to present the experiences and contextual practices of FCC from the perspectives of families and clinicians in Ghanaian NICUs. This may at least provide a dogma for future research on FCC practices in Ghanaian NICUs.

## Methods

### Study design

The researchers adopted a qualitative exploratory descriptive design [22] to study the contextual practices of FCC among families and clinicians at the NICU. The rationale for adopting this design was to explain the phenomenon of FCC from the perspectives of families and clinicians such as nurses, midwives, and doctors in the NICU [23]. Additionally, the Family Care Theory (FCT) was used as the theoretical framework [24, 25]. This offered the opportunity to study the contextual practices of FCC in the intensive care units in the Ghanaian setting. The study was conducted between July 2020 and July 2021.

### Setting

The NICUs of two public tertiary teaching hospitals in the northern and southern parts of Ghana were used for this study. These NICUs do not have any documented practices of FCC, albeit families are occasionally involved in the care via scheduled visitations. Families are not allowed to stay overnight at the NICUs in Ghana. These neonatal units have open cubicle wards with optimum logistics and separate kangaroo care rooms that admit all kinds of neonatal cases including birth asphyxia, birth injuries, neonatal jaundice, preterm, congenital abnormalities, and sepsis. These babies are medically and surgically managed by mainly general doctors, registered nurses, registered midwives, and a few specialists in neonatal care, paediatrics and intensive care. The cumulative bed capacity of the two NICUs used in this study was 105, although they often admit neonates to almost double their primary capacities.

### Participants

The study recruited families and clinicians as participants. Families, as defined in this study, included a father, mother, senior sibling, child and significant other who is caring for a critically ill child in intensive care. Clinicians in this study refer to nurses, midwives and doctors currently stationed at the NICUs. Inclusion criteria for the family were father, mother, senior sibling (more than 18 years old), and significant other with a critically ill child who has stayed at the ICU for more than 24 h. The inclusion criteria for clinicians were nurses, midwives and doctors who have at least 2 years of working experience in neonatal intensive care. The participants recruited for the interviews were different from those in the focus group discussions. This was done to guarantee that data on the study phenomenon was gathered from a variety of perspectives.

### Data collection

The approved ethics and introduction letters were used to seek permission from the departmental and unit managers at the NICUs of the two settings. The head nurses (matrons) of the NICUs assisted the researchers in identifying clinicians who met the inclusion criteria of the study. Similar arrangements were made by the head nurses to sample families who stayed at least 24 h in the ICU. Subsequently, the participants were provided information sheets and informed consent, with additional consent for a voice recording if they agreed to participate. For data triangulation and to acquire diverse perspectives from the participants, the researchers used both interviews and focus group discussions as techniques of data collection. The first author conducted the interviews and FGDs with the help of six trained research assistants. The interviews and FGDs were conducted in a secured room at each setting provided by the head nurses of the ICUs. This was necessary to avoid interference as the data was audiotaped. The researchers purposefully chose 84 participants to take part in 24 individual interviews and 12 focus groups with five participants in each group. Each FGD composed of five family members or clinicians and lasted for at least 45 min. Each individual interview lasted for at least 30 min. The rationale for the collection of extensive multiple forms of data was to obtain thick data to provide a vivid description of the FCC phenomenon in the Ghanaian NICUs. Table 1 provides further details of the qualitative data collected for this study.

**Table 1:** Details of qualitative data collected

Qualitative Data	Family		Clinicians		Total
	**Hospital T**	**Hospital K**	**Hospital T**	**Hospital K**	
Individual interviews	6	6	6	6	24
Focus Group Discussions (5-members)	3	3	3	3	12
Total transcripts	9	9	9	9	36
Total participants	21	21	21	21	84

Hospital T= Tamale Teaching Hospital, Hospital K= Korle Bu Teacing Hospital

### Data analysis

The researchers transcribed all the data into English text with the help of language experts, where the interview language was the local dialect (Dagbanli and Twi) of the participants. All transcripts were read severally by the researchers to check grammar and to get immersed in the data. To ensure anonymity and confidentiality, codes were attached to the transcripts, the setting and the participants. In this study, hospital K was assigned code K and hospital T was coded as T. Interviews were coded as I, FGDs were coded as D, families were coded as F and clinicians were coded as C. Serial numbers were added to these codes to generate the anonymous codes. The researchers employed open-inductive content analysis to extract fragmented data from the transcripts by comparing data transcripts and examining them for similarities, differences, and patterns. The initial codes were kept close to the participants’ words, all in a bit to ensure methodological rigour [26]. The researchers then used focused coding to refine and theoretically sample the fractured data into groups by selecting key terms that subsume the initial codes created from the data. Finally, the researchers reflected on the categories emerging from the data to integrate and weave all the fractured data together to arrive at the contextual perspectives of the participants on the practices of FCC in the Ghanaian NICUs. These iterative content analysis processes were facilitated by importing all the 36 transcripts into MAXQDA version 2020 qualitative software. The lexical search, word clouds, comparison of retrieval patterns and code/sub-code model functions of the qualitative software were employed to conduct the analysis.

### Ethics approval and consent to participate

Ethical clearance was obtained from the two Institutional Review Boards (TTH/R&D/127, and STC/IRB/000145/2019). The research was conducted in accordance with the institutional ethics guidelines of the Tamale Teaching Hospital and the Korle-Bu Teaching Hospital as well as standard ethical practices for conducting research. An information sheet was provided and informed consent was sought from the participants as well as additional consent for voice recording. The audio tapes have been kept under lock and key and have only been used for this study.

## Results

### Participants’ backgrounds

A total of 84 participants were recruited. They comprised of 42 (50%) families, 29 (43.5%) nurses/midwives, and 13 (15.5%) doctors. A total of 24 individual interviews and 12 FGDs for both families and clinicians were conducted. The ages of the families ranged from 18–54 years, with an average age of 31 years. Most of the family participants (75%) had at least a senior high school education, with about 42% of them having tertiary education. The clinicians were between the ages of 26 and 52 years with an average age of 38 years. The clinicians had a minimum of 5 years of working experience and a maximum of 26 years of working experience.

### Categories and sub-categories

Three main categories emerged from the data: contextual practices of FCC, family experiences and ICU clinicians’ experiences of FCC practices.

### Contextual practices of FCC

This data produced three sub-categories to illuminate the participants’ perspectives of FCC practices in Ghanaian NICUs: respect & dignity, culture and religion and multidisciplinary approach. FCC in this context is partly practiced and refers to shared decision-making and respect between families, clinicians and managers using a multi-disciplinary approach to prioritize the provision of quality and satisfactory childcare.

#### Respect and dignity

To practice FCC, clinicians must *respect* families and strive to *understand their perspectives* on care. Respecting the opinions of families will promote their *participation* in ICU care. The approach to families should *preserve* their *dignity* since the *sickness* of a child *can cause disorientation.* However, some clinicians did not accord some families the *rightful respect* due to such families’ poor sociocultural backgrounds.

#### Culture and religion

According to the participants, families should be accepted into ICUs regardless of their race, ethnicity or cultural background. Some families will refuse treatment for their children, such as blood transfusions because of their religious beliefs. In this context, the gender of a child had a substantial impact on most families' participation in the care process. Due to their cultural preference for male children over female children some families may abandon their female infants at the hospital and such practices may have an impact on the family's full participation in care. As a result, the participants stated that before FCC can be implemented, clinicians should endeavour to learn and accept the cultures and religions of their patients and families.

#### Multidisciplinary approach

Participants touted FCC as an important concept in child health that must employ a multi-disciplinary approach, including community health nurses (CHN) and social welfare in the provision of childcare in the ICUs. The CHNs, for example, will promote continuity of care through the identification of community support groups for discharged patients from the ICUs. This will provide family support and integration of the sick child into the family and community, especially for those with congenital abnormalities. However, there is a gap in FCC practices as clinicians seldom involve families in decision-making about childcare in the Ghanaian ICUs. This was due to the challenging nature of ICU services even though the clinicians recognized the importance of involving the families in decision-making about the care of their critically ill children in the ICUs.

### Family experiences

This particular category had three sub-categories: emotional stress, lack of information and coping strategies.

#### Emotional stress

Anxiety and emotional stress were common among families with seriously ill children hospitalized in the intensive care unit. "It feels empty," "very anxious," and "very awful," they said, describing their experiences. The phenomenon of going home to sleep without the child generated this anxiety: "you can't sleep," "you feel like your baby is crying," and "boxes are carried with dead babies."

#### Lack of information

The absence of an open flow of information and feedback on the processes and progress of their children's illness were unpleasant experiences according to the families. This hampered their ability to fully participate in ICU care and exacerbated their anxiety. Families in the intensive care unit (ICU) desire more information about the care process to feel more at ease.

#### Coping strategies

Prayers, crying, taking time off work and receiving occasional assistance from the staff were among the coping strategies used by families to deal with the anxiety associated with the ICU phase of treatment. Even though some mothers grieved as a result of these events, crying was also a way for them to vent and deal with the stress. The following are some of the participants' accounts of their experiences:

### Clinicians’ experiences

The experiences of the clinicians relative to the practices of FCC in the Ghanaian ICUs were described by three sub-categories: support, counselling and education as well as funding challenges.

#### Support

The clinicians narrated that families need the support of staff and the system to deal with the stress of participating in the care of their critically ill children in the ICUs. Most mothers go through caesarean section and are often left alone to do laboratory and pharmacy errands. The staff usually assists the families to meet these needs. In our case, some families were helpful in caring for their critically ill children, while others were not. Some anticipated early discharge regardless of the outcome of their children's condition, which is challenging for the care providers. Such families sometimes requested discharge against medical advice (DAMA) because the mothers were left alone to support the care with limited resources to sustain their stay at the ICU:

*"… My experiences with mothers in the care of their newborns have been okay; some are ready and supportive, and some are not supportive in the care of their newborns…”* ***TCI2.*** *"You can just imagine a mother who has just delivered,  probably through a caesarean section. Her husband has to be away to work and bring money. So, she is left alone with no sister and no support”* ***KCI1*** *. “The mothers are left alone to take care of the baby… the issue that you will encounter is that the mother is looking to be discharged irrespective of the baby’s state of health because of a lack of resources to take care of the baby.”* ***TCI6.***

#### Counselling and education

There were counselling rooms used for sharing information with the families at the NICU. Families were counseled by clinicians by reassuring them and explaining the child's condition to them. The counselling sessions were also used to educate families and answer all their questions to allay anxiety. Due to personnel challenges, this was not done for all families admitted to the intensive care unit. The clinicians, however, indicated that permanent clinical psychologists in the ICU would be helpful in addressing the emotional stress on both families and clinicians. The clinicians educated families on the clothing of the babies, feeding the critically ill neonates and maintaining the neonates’ body temperature via Kangaroo Care and extra clothing.

*"We have a counselling unit and every patient who comes goes through a first-time counselling session…we explain to them the processes here and their children’s condition”* ***KCD3E*** *.": .... we need a clinical psychologist in the ICU because the ICU is a very stressful place, hence the role of a clinical psychologist cannot be under emphasized...”* ***KCI1*** *.*

#### Funding challenges

Clinicians intimated that in the ICUs, services are very expensive. This challenged the provision of care, especially for teenage parents and those with congenital abnormalities requiring prolonged NICU stays. They recommended strengthening institutional-based funding, including *social welfare* and setting up a national child health fund to support such needy families.

*“Per expert assessment and interaction with families especially those who come with congenital issues, it is identified that they have challenges like financial issues…”****TCD1C****. “… intensive care is expensive and is quite a new concept in this part of this country, where they are not able to afford the care and people don’t really appreciate that a lot of resources are required to take care of babies”****TCI6."****…the social welfare should work, because some of the parents are teenagers who cannot fend for themselves let alone their critically ill children” ****KCI4****"Our facility has a fund for child health support, contributed by clients who come to the facility and some other donations, but that fund is inadequate to meet the financial needs of families’ especially needy families with critically ill children requiring intensive care, because it’s very expensive. We need a national fund to support the family in the care of their critically ill children” ****KCI5***. *"We could have funds where there will be donations for us to use to support the needy ones”****TCI4.***

## Discussions

FCC in the Ghanaian context is partly practiced at the NICUs. Participants defined FCC as shared decision-making and respect between the clinicians, managers, and families using a multi-disciplinary approach and education to prioritize the provision of quality childcare regardless of the family’s sociocultural background. The shared decision-making aspect of the finding is consistent with the philosophy of FCC in the Canadian [27] and Norway [11] contexts. In the work of WHO, decision making and patient satisfaction are essential components of ensuring quality health care [28]. Conceivably, the increased ICU admissions, with their attendant workload on clinicians, inadequate spaces and personnel in the Ghanaian NICUs may be accountable for the occasional involvement of families by clinicians in decision-making. Health managers, the Ghana Health Services (GHS) and the Ministry of Health (MOH) must take immediate steps to build more NICUs across the country and expand as well as equip the existing ICUs with adequate logistics and specialized personnel.

The clinicians restricted the definition of FCC in the ICU to only the father and mother of a sick child and therefore granted access to them alone. Yet, respectful and dignified care [1] in our context is considered to be care that accepts all family members, irrespective of their sociocultural backgrounds. Contrary to these findings, the acceptance of all family members, including extended family into Danish ICUs improved health outcomes for preterm babies [19]. Perhaps, this finding in the Ghanaian setting was attributed to a lack of space and personnel at the ICUs to fully engage families as experts as proposed by the FCC concepts [1]. Maybe they would have attached more meaning to it if the bottle necks of space and inadequate personnel were removed. This suggests that health professionals, managers and institutions must create the needed spaces and train the professionals to build collaborative partnerships with families of sick children in the ICUs in making decisions on the care processes. In that way, quality care may be guaranteed as the families may feel involved and satisfied with the care.

The study findings also advocate for a multi-disciplinary approach towards FCC practice in the Ghanaian context. A multi-disciplinary approach to FCC has been recommended in a qualitative study conducted in Finland [12]. Possibly, the participants recognized the diverse roles played by different professional groups in supporting families in NICU care. This means the implementation of FCC in the health system should be approached by all stakeholders, including clinicians (e.g., nurses and doctors), managers (e.g., directors and administrators), specialists (e.g., clinical psychologists and community/public health nurses), and institutions like MOH, GHS, and Health Facilities Regulatory Authority (HeFRA). Thus, the implementation of FCC practices should not only be left in the hands of nurses and doctors, but all these parties must be fully integrated into it to be successful. Community health nurses, for example, can be stationed in the NICU to follow up with critically ill children and their families after discharge via home visits. Furthermore, the inclusion of community health nurses in NICUs will help immunize babies and those with non-immune-related conditions under observation to prevent vaccine-related diseases. Similarly, a clinical psychologist in the ICUs will help both clinicians and families manage the emotional and psychological stressors, including anxieties associated with the ICU phase of care. By so doing, the health system will be in a better position to address the non-pathological aspects of maternal and child health that may affect their physical health. This may also help to improve the overall health and well-being of ICU patients' families and clinicians. Additionally, the study revealed that clinicians are willing to accept and support families to fully participate in the NICU phase of care. Clinicians recognise the significant roles families play in the NICU, for example in providing information and purchasing medicine and supplies to support the care of their critically ill loved ones. This finding resonates with the concept of FCC practiced in Japan [29]. However, families were seen to be helpful in complementing the efforts of clinicians in the NICUs, in most cases, to provide quality care at their best. Thus, clinicians are not against the policies on the concepts of FCC practices in the ICU. The clinicians showed empathy to families with critically ill children in the ICU. But, they maintained that the provision of funding for intensive care units will facilitate the successful integration and implementation of FCC into the ICU since it will, for example, require more space and strict protocols of infection prevention and control for both parties.

Families with critically ill children in the ICUs described their own experiences as being anxious [30], awful, and painful. This finding buttressed the initial call for a multi-disciplinary approach to FCC practice where the services of a clinical psychologist, including professional counselling, can be provided for families to manage their anxieties. Further research might focus on monitoring the quality of ICU care and evaluating client satisfaction in our context to better appreciate the drivers of quality care, synchronize best practices across settings and harness the benefits of FCC.

### Strengths and limitations

The study draws its strength from the multiple forms of interviews and focuses on group data collected to arrive at the findings presented. However, our findings are limited in their qualitative nature and also by the fact that the data was collected from only the NICU setting, but the inclusion of 24 interviews and 12 FGDs resulted in data saturation. Again, data collection from different settings could have enriched the findings. Some of the family data were collected in local languages and translated into English with the assistance of a language expert; this may have resulted in some data loss.

## Conclusions

Family-Centered Care is shared decision-making and respect between the clinicians, managers, and families using a multi-disciplinary approach and education to prioritize the provision of quality neonatal care regardless of the person’s sociocultural background. Interventions such as emotional support via counselling by a certified clinical psychologist, regular information from staff, and funding support from the government may help families cope with the NICU phase of care and build their confidence for continuity of care. Future research should apply multiple methods to study FCC in different settings of child health practice to help harmonize best practices for improved child health and family outcomes.

## Data Availability

The data set and materials used for this study are accessible upon reasonable request from ASA, the principal investigator.

## Abbreviations

FCC: Family-Centered Care
IPFCC: Institute of Patient and Family-Centered Care
ICU: Intensive Care Unit
CHN: Community Health Nurses
NICUs: Neonatal Intensive care Units
FGDs: Focus Group Discussions
MOH: Ministry of Health
GHS: Ghana Health Services
HeFRA: Health Facilities Regulatory Authority
WHO: World Health Organisation
